# A Nationwide Virtual Research Education Program for Medical Students in Pakistan: Methodological Framework, Feasibility Testing, and Outcomes

**DOI:** 10.3389/fpubh.2021.812130

**Published:** 2022-01-10

**Authors:** Ali Aahil Noorali, Maha Inam, Hamna Shahbaz, Hareem Rauf, Faiqa Binte Aamir, Farah Khalid, Saadia Abbas, Abdullah Saeed, Muhammad Daniyal Musharraf, Asma Altaf Hussain Merchant, Babar S. Hasan, Muneera A. Rasheed, Fyezah Jehan, Muhammad Tariq, Adil Hussain Haider

**Affiliations:** ^1^Department of Medicine, Medical College, Aga Khan University, Karachi, Pakistan; ^2^Health Data Science Center, Clinical and Translational Research Incubator, Medical College, Aga Khan University, Karachi, Pakistan; ^3^Dean's Office, Medical College, Aga Khan University, Karachi, Pakistan; ^4^Medical College, Aga Khan University, Karachi, Pakistan; ^5^Department of Surgery, Washington University in St. Louis, St. Louis, MO, United States; ^6^Department of Paediatrics, Medical College, Aga Khan University, Karachi, Pakistan; ^7^Department for Educational Development, Medical College, Aga Khan University, Karachi, Pakistan; ^8^Department of Surgery and Community Health Sciences, Medical College, Aga Khan University, Karachi, Pakistan

**Keywords:** virtual course, research teaching, feasibility, medical students, public health

## Abstract

**Introduction:** Equipping young medical trainees with fundamental research skills can be a promising strategy to address the need for professionals who can understand and responsibly communicate evolving scientific evidence during a pandemic. Despite an ardent interest to partake in research, most educational institutions in Pakistan and other low-middle income countries have not yet adopted a comprehensive strategy for research skills education. The authors aimed to design and assess the feasibility of implementing the first nation-wide virtual research workshop for medical students in Pakistan.

**Methods:** The course “Beginners Guide to Research,” designed as a nation-wide virtual research workshop series, was conducted for medical students across Pakistan in June 2020. Four interactive live workshops took place online on alternate days from June 22nd, 2020, to June 27th, 2020, each lasting 1–2 h. Outcomes included: (i) reach, (ii) efficacy as indexed by pre-post change in score pertaining to knowledge and application of research and (iii) self-rated perceptions about understanding of research on a Likert scale.

**Results:** 3,862 participants enrolled from 41 cities and 123 institutions. Enrolled participants belonged to the following provinces: Sindh (*n* = 1,852, 48.0%), Punjab (*n* = 1,767, 45.8%), Khyber Pakhtunkhwa (*n* = 109, 2.8%), Azad Jammu and Kashmir (*n* = 84, 2.2%) Balochistan (*n* = 42, 1.1%). We also saw a few registrations from international students (*n* = 8, 0.2%). Mean (SD) age of enrolled medical students was 21.1 (2.1) years, 2,453 (63.5%) participants were female and 2,394 (62.0%) were from private-sector medical colleges. Two thousand ninety-three participants participants filled out all four pre-test and post-test forms. The total median knowledge score improved from 39.7 to 60.3% with the highest improvements in concepts of research bioethics and literature search (*p* < 0.001) with greater change for females compared to males (+20.6 vs. +16.2%, *p* < 0.001) and private institutions compared to public ones (+16.2 vs. +22.1%, *p* < 0.001).

**Conclusion:** The overwhelming enrollment and significant improvement in learning outcomes (>50% of baseline) indicate feasibility of a medical student-led research course during a pandemic, highlighting its role in catering to the research needs in the LMICs.

## Introduction

Responsible and effective scientific communication has been one of the key lessons during the pandemic (1). Such communication requires a sound understanding of science, epidemiology, and research in medicine (2). This communication is led by experts and is informed by incoming scientific evidence, which is crucial to disseminate appropriately to the public in a time of crisis like the pandemic and during interactions with patients otherwise. However, over the last three decades, the percentage of physician-scientists has plummeted with numbers going down from 4.7% in the 1980s to 1.5% in the 2010s in the US alone (3, 4). While evidence is lacking for a low-middle income country such as Pakistan, there are only a handful of physician-scientists in the country, which can be attributed to an lack of skills, knowledge, mentorship, and funding support leading to poor understanding of research causing misinformation (5, 6).

To address this challenge and reverse this trend, it is necessary to develop and encourage a research potential early on in budding physician-scientists. This is of pronounced importance in settings like Pakistan, which are plagued with a lack of adequate public health training institutions, lower rates of literacy in the general population and an overall mistrust in the profession of medicine. It is critical to develop and encourage an understanding of scientific research potentially early on in emerging physician-scientists during their medical training (7). This skills development lies at the forefront of the information age, where wide access to evidence-based practice and bench-to-bedside translational research has already led the paradigm shift in teaching and learning (8). Thereby, developing equitable mechanisms for resource-limited settings to equip trainees with research skills is imperative. If left unattended, this deficit will only widen the divide and disparity in science and medicine.

While the importance of developing these research skills within medical students has been well-outlined, many universities in Pakistan have not yet adopted a comprehensive curricular strategy which could have come in very handy during the pandemic (9). Aligning with recent calls for medical students to be involved in the pandemic response (10, 11), the Student Research Forum, an autonomous research body at a private medical university decided to implement an intervention for medical students. The intervention was conceptualized to aid bridging the gap of evidence-based communication. In this paper, we first describe the process our team underwent to design and implement the intervention. We also evaluate the feasibility of this intervention.

## Methods

### Study Setting

The Student Research Forum (SRF) is a student-run and faculty-supervised research organization registered at the Aga Khan University. The study setting included SRF, the university administrative departments and the SRF Student Ambassador Network. Medical colleges in developing countries such as Pakistan have only recently begun to dedicate their resources toward promoting research within the student community (12). South Asia contributes to only 1.2% of research articles despite being one-fifth of the world's population (13). A cross sectional study conducted in Pakistan showed that a majority of the medical students from either public or private sector universities were keenly interested in medical research and 91% expressed an interest toward having medical research a part of basic medical education (9).

### Study Design

A nation-wide virtual research workshop series conducted for medical students across Pakistan was designed as a feasibility study (14, 15). The main objectives of feasibility included (i) the assessment of recruitment capability and resulting sample characteristics, data collection procedures and outcome measures, (ii) acceptability of the intervention and study procedures, resources, and ability to manage and implement the study and intervention, and (iii) preliminary evaluation of participant responses to the intervention (16). Objective outcomes included change in scores (pre-test to post-test) pertaining to knowledge about key research topics and application of research methods. Subjective outcomes involved changes in perceptions toward research on a standard Likert scale.

### Sampling Strategy and Study Enrollment

A triphasic enrollment strategy was deployed, which involved marketing and communications on three concurrent fronts: social media (Facebook, Twitter, and Instagram) of SRF. Registrations were done using a Google Form link for 2 weeks prior to the workshop series. The online workshop series was freely accessible to all registered participants, who were provided de-identification codes for the assessment study. Submission of both pre and post questionnaires contributed toward the eligibility criteria for inclusion in analysis. This study was approved by the institutional review board (AKU ERC 2020-5131-11751). Digital informed consent was obtained from all students at the time of enrollment.

### Intervention Design and Implementation

**Figure 1:**
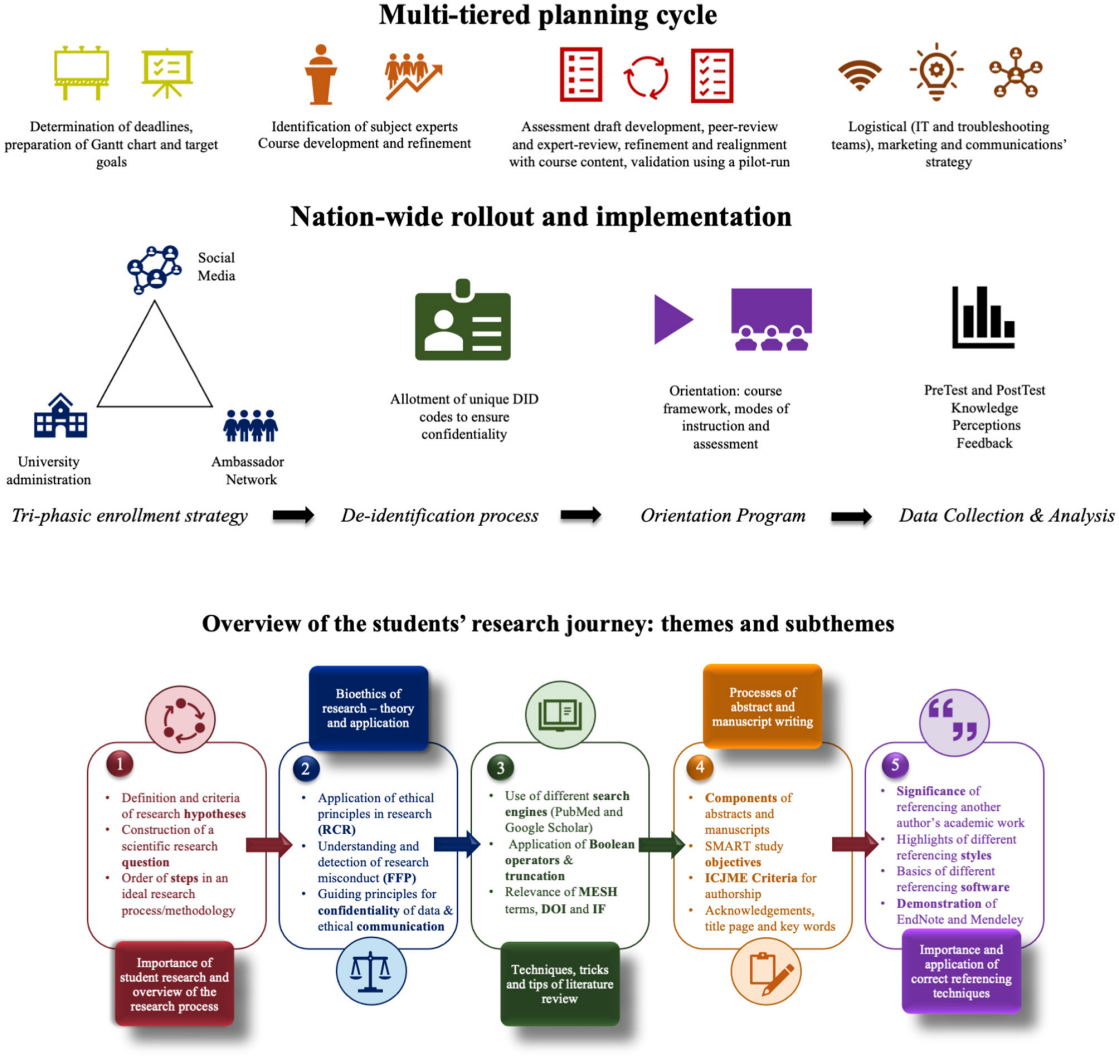
Snapshot of the multifaceted design, implementation process and an overview of the students' research journey.

“Beginners Guide to Research” was a nation-wide virtual research workshop series conducted for medical students across Pakistan. The strategic planning cycle (Figure 1) focused on course development and refinement, identification of subject experts in the university and development of the logistical and information technology infrastructure. Four interactive workshops were planned, each with specific learning outcomes that were predesigned and scrutinized by experts in research methodology (Table 1).

**Table 1:** Summary of the key objectives and format of instruction and assessment employed in the research skills development courses.

	**Brief course content theme**	**Outline and objectives**
1.	Importance of research and overview of its components	To discuss the importance of research in the medical community and an overview of its components (research question, literature search and referencing, data collection and analysis, abstract and manuscript writing).
2.	Bioethics of research	To impart an understanding of key terms in bioethics of research (plagiarism, reliability and accuracy of results, consequences of fabrication, importance of IRB approvals).
3.	Importance of literature review and methods of conducting it using different resources	To highlight the purpose of a literature review and the role of authenticity when referencing scholarly work in research papers; To provide an overview on how to conduct a literature search effectively, using various resources such as PubMed and Google Scholar.
4.	Format and components of abstracts and manuscripts	To discuss the format of a manuscript, key ingredients of a robust manuscript, types of abstracts and connecting abstracts and manuscripts
5.	Need for and importance of referencing Application of research management software used in referencing	To underline the need for and importance of duly referencing another author's work; To showcase different styles of referencing most commonly used by journals; To demonstrate and practice referencing software: EndNote and Mendeley

The workshop series commenced on the 22nd of June 2020 and continued until the 27th of June 2020. The workshop series consisted of four sessions conducted online using Zoom application Version: 5.0.4 (25694.0524) and Live-Streaming Webinar (Video IBM Channel). Figure 1 comprehensively details the research journey that each individual student underwent throughout the series, with a succinct description of the pertinent learning outcomes and sub-themes. An orientation program ensued to give participants an overview of the course framework.

The duration of each workshop was between 1 and 2 h, which was the duration pre-selected to find the balance between providing speakers enough time to explain basic concepts and the audience adequate and digestible content that did not exceed their online attention span. Concepts outlined in the workshop were kept to a beginner's level of understanding.

### Interaction and Communication

To make the workshops interactive and to enable brisk communication, the entire cohort of participants was divided into 16 groups and each group was appointed a team-lead from the organizing team, along with a few other members for troubleshooting and communication purposes. Two platforms were maintained and monitored for incoming questions and correspondence: “Backchannel Chat” and a dedicated email address. Both these platforms were operational in real-time and for the duration of the workshop. This “many-to-some-to-one” strategy enabled participants to reach out to the central team and, by extension, the presenter.

Additionally, some workshops also employed techniques for the session presenters to interact with the audience using the “one-to-many” format by conducting live practice-based demonstrations. For example, the session on “Literature Search” included a live demonstration on PubMed, whereby the participants could both observe the presenter conducting the search in real-time, and simultaneously perform the same on their end also.

### Data Collection Tools

The study was evaluated for two domains of feasibility: reach and preliminary efficacy (17). Outreach was determined using the total number of registrations, institutions, and cities across Pakistan.

The questionnaires to assess efficacy had two components:

1. Test Section: This included both scenario-based application of basic research concepts and knowledge-based assessment of fundamental terms and principles. The testing approach was Best Choice Questions (BCQs). Pre-test and post-test questionnaires evaluated participants on their knowledge of research and workshop session objectives, as well as application of concepts. The questions were designed as multiple-choice questions including conventional multiple choice and scenario-based questions.
2. Participants' perceptive rating section: This aimed to evaluate the participants' own perception of their understanding of research sub-topics corresponding to each session assessed on a self-rating scale. A content-relevant Likert's scale was developed to evaluate this, whereby “5” was equivalent to strong agreement with the prompt and “1” was equivalent to strong disagreement. Similar to the test section, participants' perception rating was also collected before and after the workshop.

To ensure reliability of the questionnaire, a pilot run of each test was also conducted. Medical students who were not part of the workshop organizing team and were not registered participants of the workshop were selected for a trial run of the questionnaires from a pre-test perspective. The pilot run was successful and did not require any significant amendments to the questionnaires.

### Statistical Analysis Plan

Analysis was done using StataCorp. 2019 (Stata Statistical Software: Release 16. College Station, TX: StataCorp LLC). After the initial registrations were procured, each participant was allocated a unique De-Identification code (D-ID). This step was important to maintain participants' confidentiality and to reduce the risk of researcher bias. To ensure validity and accuracy, only those who filled both pre-test and post-test of a particular workshop were included in the final analysis. Pre-test and post-test scores were calculated overall, for each workshop and for eight distinct categories. Since the data were skewed, median and interquartile range (IQR) were used as a measure of central tendency, and change in medians were calculated for each of the above. The significance in change of scores was calculated using Wilcoxon signed-rank test, and *p*-values <0.05 were statistically significant.

## Results

### Reach

The series saw enrollments from a total of 3,862 participants from 41 cities and 123 institutions (Table 2). Enrolled participants belonged to the following provinces: Sindh (*n* = 1,852, 48.0%), Punjab (*n* = 1,767, 45.8%), Khyber Pakhtunkhwa (*n* = 109, 2.8%), Azad Jammu and Kashmir (*n* = 84, 2.2%), Balochistan (*n* = 42, 1.1%). We also saw a few registrations from international students (*n* = 8, 0.2%). In terms of intra-city representation, 1,701 (44.0%) of the participants came from Karachi. Two thousand six hundred ninety-one (69.7%) participants reported having no academic publishing experience prior to registering in the workshop series.

**Table 2:** Enrollments, demographics and participant characteristics (*n* = 3,862).

**Variable**	**Beginner's guide**	
Enrollments	Cities	41
	Universities	123
	Participants	3,862
Age, in years, [mean (SD)]	21.1 (2.1)	
Gender; *n* (%)	Female	2,453 (63.5)
	Male	1,365 (35.3)
	Preferred not to respond	44 (1.1)
Year of education; *n* (%)	1st Year, MBBS	903 (23.4)
	2nd Year, MBBS	872 (22.6)
	3rd Year, MBBS	684 (17.7)
	4th Year, MBBS	802 (20.8)
	5th Year, MBBS	601 (15.6)
Type of education system; *n* (%)	Public	1,468 (38.0)
	Private	2,394 (62.0)
Representation; *n* (%)	Sindh	1,852 (48.0)
	Punjab	1,767 (45.8)
	Khyber Pakhtunkhwa	109 (2.8)
	Balochistan	42 (1.1)
	Azad Jammu and Kashmir	84 (2.2)
	Outside Pakistan	8 (0.2)
Prior publishing experience (optional question); *n* (%)	Yes	140 (3.6)
	No	2,691 (69.7)
	Did not report	1,031 (26.7)

The mean (SD) age of the registered participants was 21.1 (2.1) years. Two thousand four hundred fifty-three (63.5%) participants were females, 903 (23.4%) were in their first year of medical school training and 2,394 (62.0%) hailed from the private-sector medical institutions of Pakistan.

### Preliminary Efficacy

#### Pre-post Knowledge Scores

The assessment part of the workshop was voluntary. In order of the respective workshops (1 through 4), a total of 2,718, 2,690, 2,525, and 2,480 respondents filled out both pre-tests and post-tests for respective workshops indicating slight decline per session. Overall, a total of 2,093 (54% of total registrations) participants filled out pre-test and post-test questionnaires for all 4 sessions.

**Figure 2:**
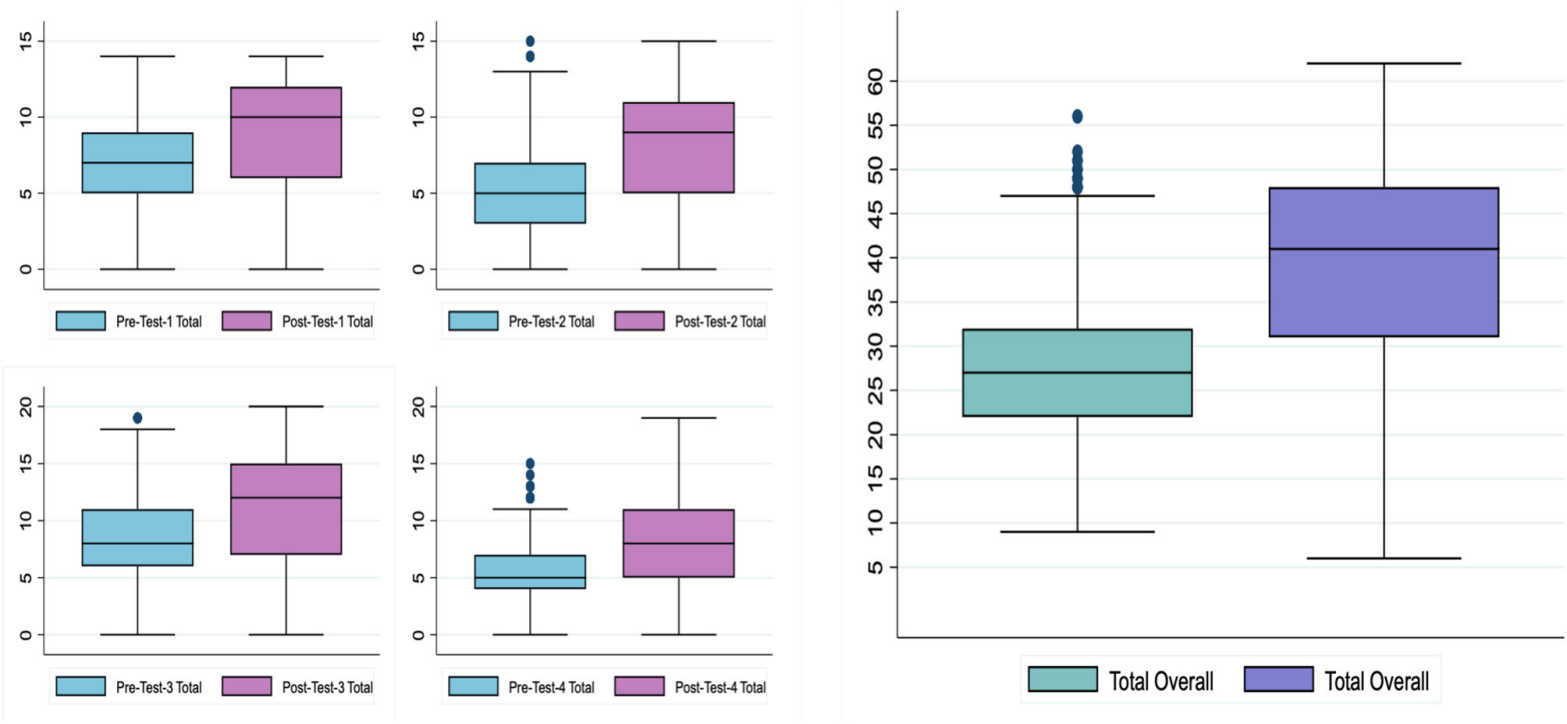
Objective assessment: box-and-whisker plots showcasing changes in total scores from baseline pre-workshop aggregates to post-workshop aggregates.

Full series' analysis (*N* = 2,093) showed that the overall median (IQR) score increased from 27 (22–32) to 41 (31–48) (*p* < 0.001) and the percentage median score, as a proportion of the maximum possible score, improved from 39.7 to 60.3% (Figure 2; Table 3). Similarly, individual workshop scores showed a positive increase in participant scores after attending the workshop (*p* < 0.001; Table 3). The workshops on literature search along with those on abstract and manuscript writing showed the highest change in scores (Δ Median = +4 for both, Δ% score = +26.7 and +20.0, respectively). The workshops on referencing and introduction to research also showed positive deflections in the participant scores (Δ Median = +3 and +2, Δ % score = +15.8 and +14.3, respectively).

**Table 3:** Comparison and univariate analysis of pre-test and post-test knowledge using Multiple Choice Questions (MCQ) format questionnaires.

**Comparison of pre-test and post-test scores**									
**Test**	**Questions**	**Responses; *n***	**PreTest median (IQR)**	**PostTest Median (IQR)**	**Δ Median^a^**	**% Pre-test score^b^**	**% Post-test score^b^**	**Δ % Score^c^**	***p*-value^d^**
Total test 1	14	2,718	8 (6–9)	10 (8–12)	2	57.1	71.4	14.3	<0.001
Category A^e^	5	2,718	3 (3–4)	4 (3–4)	1	60	80	20	<0.001
Category B	9	2,718	4 (3–6)	7 (5–8)	3	44.4	77.8	33.3	<0.001
Total test 2	15	2,690	6 (4–7)	10 (7–12)	4	40	66.7	26.7	<0.001
Category C	9	2,690	3 (2–4)	6 (4–7)	3	33.3	66.7	33.3	<0.001
Category D	6	2,690	2 (1–3)	4 (3–5)	2	33.3	66.7	33.3	<0.001
Total test 3	20	2,525	8 (6–11)	12 (8–15)	4	40	60	20	<0.001
Category E	9	2,525	4 (3–5)	6 (4–7)	2	44.4	66.7	22.2	<0.001
Category F	11	2,525	4 (3–6)	6 (4–8)	2	36.4	54.5	18.2	<0.001
Total test 4	19	2,480	5 (4–7)	8 (5–11)	3	26.3	42.1	15.8	<0.001
Category G	8	2,480	2 (1–3)	3 (2–5)	1	25	37.5	12.5	<0.001
Category H	11	2,480	3 (2–4)	5 (3–7)	2	27.3	45.5	18	<0.001
Overall	68	2,093	27 (22–32)	41 (31–48)	14	39.7	60.3	20.6	<0.001
**Univariate stratified analysis (*****n*** **=** **2,093)**									
**Variable**	**Questions**	**Responses** ***n*** **(%)**	**Pre-test median (IQR)**	**Post-test median (IQR)**	**Δ** **Median**^a^	**% Pre-test score** ^b^	**% Post-test score** ^b^	**Δ** **% score** ^c^	***p*** **-value** ^d^
Gender									<0.001
Female		1,532 (73.2)	27 (23–32)	41 (32–48)	14	39.7	60.3	20.6	
Male		549 (26.2)	27 (22–23)	38 (29–47)	11	39.7	55.9	16.2	
Not Reported		12 (0.6)	26 (22–33)	43 (24–48)	17	38.2	63.2	25	
MBBS year									0.076
1st Year	68	466 (22.2)	26 (21–31)	39 (29–46)	13	38.2	57.4	19.1	
2nd Year		540 (25.8)	27 (22–32)	41 (31–48)	14	39.7	60.3	20.6	
3rd Year		379 (18.1)	27 (21–31)	40 (32–47)	13	39.7	58.8	19.1	
4th Year		417 (19.9)	28 (23–33)	41 (33–49)	13	41.2	60.3	19.1	
5th Year		291 (13.9)	29 (23–35)	42 (32–50)	13	42.6	61.8	19.1	
Institution									<0.001
Private		1,301 (62.2)	27 (22–32)	42 (33–49)	15	39.7	61.8	22.1	
Public		792 (37.8)	27 (22–32)	38 (29–46)	11	39.7	55.9	16.2	

a *Δ Median denotes change in median which is calculated by (Post-test median – Pre-test median)*.

b *% has been calculated using (median/maximum score) ×100*.

c *Δ % Score denotes change in score which is calculated by (%Post-test median – % Pre-test median)*.

d *P-value was calculated using Wilcoxon signed-rank test (comparing pre-test median and post-test median scores) since the post-tests were left-skewed*.

e *Category A, Research question, hypothesis, steps and communication; Category B, Bioethics of research; FFP, confidentiality; Category C, Concepts important to literature search, search engines, MESH, DOI and IF; Category D, Application of literature search techniques; Category E, Abstract writing and SMART objectives; Category F, Manuscript components and writing, authorship and acknowledgments; Category G, Concepts important to referencing - significance, styles and softwares; Category H, Application of referencing on EndNote and Mendeley*.

Analysis by categories showed that the highest improvements in scores were observed in bioethics of research and concepts of literature search (Δ Median = +4, Δ % score = 33.3 for both). Concepts and application of referencing showed the least improvement in scores (Δ Median = +1 and +2, Δ % score = +12.5 and +18.0, respectively).

We found that both females and males started at the same baseline median scores, but females showed a significantly higher improvement in scores (Δ Median = +14 vs. +11, Δ % score = +20.6 vs. +16.2, *p* < 0.001). Similarly, students at both public and private institutions started at the same baseline scores but the latter performed significantly better in terms of score improvement (Δ Median = +11 vs. +15, Δ % score = +16.2 vs. +22.1, *p* < 0.001). Although the year of education did not show significant differences in improvement between the categories, an increasing trend in both baseline pre-test and post-test scores was noted as the year of education increased.

#### Pre-post Self-Rating Scores

**Figure 3:**
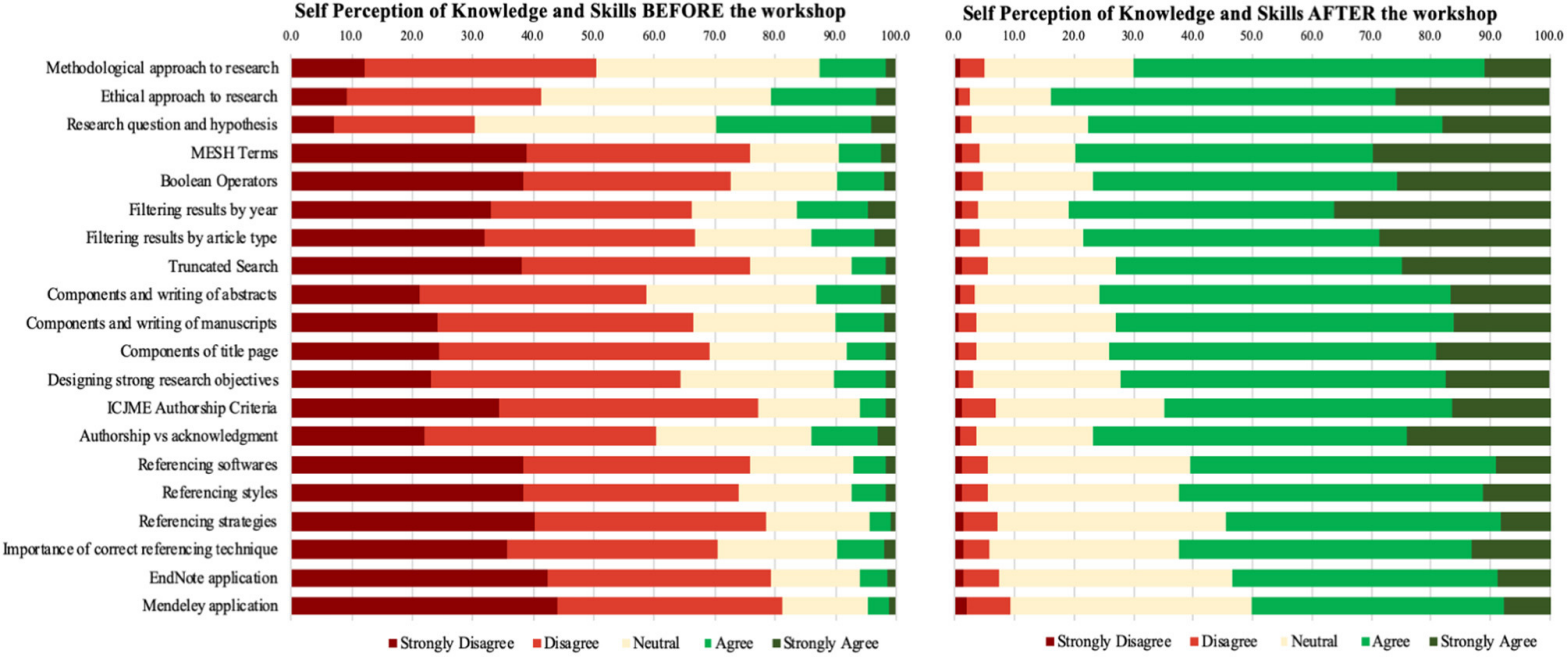
Subjective assessment: categorical assortment of participants' self-perceptions.

On the self-rating scale, the greatest overall red-to-green shift from disagreement (dark red and red) to agreement (dark green and green) was observed for the second (literature search) and third (abstract and manuscript writing) workshops (Figure 3). Similarly, within the sub-themes, the largest red-to-green shift was appreciated in perceptions about Medical Subject Headings (MESH terms) and Boolean operators (both part of the second workshop), whereas the smallest corresponding shift was seen in “research question and hypothesis” and “methodological approach to research,” (both part of the first workshop). Each item showed a positive trend from strong disagreement (dark red) and disagreement (red) to agreement (green) and strong agreement (dark-green).

## Discussion

Our main findings primarily indicate the feasibility of a large-scale national virtual research workshop designed for medical students. The workshop attracted 3,862 registrations across 123 different institutions. Assessment of 2,093 participants indicated an appreciable improvement in overall participant knowledge scores from 39.7 to 60.3% (increase was >50% of baseline) over the course of the series. To our knowledge, this is the first paper to introduce a virtual nation-wide research workshop for medical students in the region in response to the pandemic.

Participants attending our workshop had a baseline assessment score of 39.7%. Many other interventions have implemented and evaluated research skills training at local and regional levels (18–22). A cross-sectional study conducted in India showed the impact of a “Mentored Student Project” in fostering a positive attitude toward student research (23, 24). Within Pakistan, a single-center study in 2006 reported the baseline mean knowledge and attitude scores of medical students regarding research to be 49.0 and 53.7%, respectively (25). Similar to our findings, this study also reported a higher baseline knowledge score on items pertaining to literature search on MEDLINE (60.9%), components of manuscripts (60.4%) and writing of introduction (57.8%), compared to lower scores on themes pertaining to scientific hypothesis (29.4%) and characteristics of research science (23.9%) (25). Another cross-sectional study conducted on 184 medical students in Jeddah, Saudi Arabia reported the cumulative scores for students' knowledge and attitudes regarding participation in scientific research, to be 57 and 76%, respectively (26). The baseline knowledge shown in these studies and our findings collectively demonstrate how critical the need is for a strategically planned research curriculum in the region. However, understanding the baseline status-quo should not be left unattended, but rather be the foundation for a rigorous research program implementation and analysis of post-intervention outcomes.

Due to limitations imposed by the COVID-19 pandemic, our research team leveraged the concurrent ongoing switch to digital technology. Within Pakistan and beyond, there has been a quick uptake of online educational media such as Zoom, Google Hangouts and Skype (27–31). While digital learning and virtual platforms did exist before the pandemic, their use has been small-scale, unstructured, sparse, and unsustainable. Given the rapid uptake of these platforms with the onset of the COVID-19 pandemic, it was possible to reach out to and connect with a large number of participants in real time. It also ensured that costs of transportation, accommodation and logistics to another city were no longer an impediment. This ensured that the workshop remained accessible and free-of-cost to the participants, which further increased the turnout.

Online delivery of educational content brings forth a multitude of advantages, predominantly cost-effectiveness, scalability, replicability and increased accessibility (32–35). Multiple reports, including those from Pakistan, have demonstrated strong support for online learning (36–38), while others have suggested that traditional modalities of learning may be more effective in enhancing knowledge outcomes (39, 40). While it does not compare the two, our study does resonate with the support expressed for online teaching-learning mechanisms. This is seen particularly by the high response rate, significant improvement in key learning metrics and the feedback received throughout the series. Further downstream, post-pandemic, it may be useful to compare the outcomes of such an intervention between virtual and traditional platforms.

An interesting finding from our study was that a large majority of participants in the workshop were females (*n* = 2,453, 63.5%) and scored significantly higher than their male counterparts (Δ % score = +20.6 vs. +16.2, *p* < 0.001). This is different from baseline snapshots in Pakistan (25) and Saudi Arabia (41), which have reported male medical students to have a higher attitude score toward research. In contrast, a recent assessment of medical research perceptions amongst medical students in Beirut, Lebanon reports female medical students to have a more positive perception (42). The higher enrollment and better percentage improvement in female students further indicates the need to sustain this drive and to work on developing a greater interest toward research in their male counterparts.

Furthermore, a large majority (*n* = 1,852, 48.0%) of medical students belonged to the province of Sindh, particularly from Karachi. This can partly be explained by the fact the course was led by a medical school within the city. Also, the city, Karachi has a higher literacy rate (~75%), which is one of Pakistan's largest metropolitan cities (43). Medical students from Punjab, the most populous province of Pakistan, also participated in high numbers (*n* = 1,767, 45.8%). Data also show that the highest number of registered medical colleges (53%) are from Punjab (44). However, we observed that our enrollments did not significantly reach the less advantaged provinces in other parts of Pakistan like Balochistan where literacy rate is below 10% (43). This serves as a call-to-action, accentuating that a lot more needs to be done to increase access and ensure equity in such programs.

Although our workshop was the first intervention of its kind during the pandemic, the curriculum content and teaching-learning pedagogy were carefully developed and curated by experts in academic medicine. In order to attract a wider target audience from varying diasporas across the country and to transcend geographical barriers, the workshop series was deployed on a virtual platform and was kept free-of-cost. This ensured that a student's socio-economic status did not impact their motivation to partake in this learning opportunity. Planning and strategy development were very much emphasized to ensure that the learning and feedback tools were easily comprehensible to a diverse participant population. In the setting of the COVID-19 pandemic, this novel intervention enabled students, experiencing varying levels of detachment from their institutional educational curriculum, to connect and learn together in the pursuit of acquiring new skill sets. It also ensured that students could productively use their free time, while away from formal, in-person, educational programming. Since research education already faces deficits in medical education curricula, this opportunity guaranteed that students were not disadvantaged due to the pandemic, by further reductions in the training of these important skills.

A major strength of the study was led by medical students themselves at a private medical school in the country. We believe this is an example of student leadership during the pandemic apart from their skill set to be utilized in healthcare to designing courses for peers to further research understanding. The study is encouraging in the sense that medical students in LMIC can also be engaged as a workforce similar to high-income countries (45, 46).

There were similar limitations of the study. A major limitation to our study was that despite a large number of workshop participants (3,862), only 2,093 ultimately chose to be part of the assessment study and filled out all eight pre-tests and post-tests. Since the tests were only open at specific intervals before and after the workshop, this can be partly explained by logistical or technical issues that some participants may have encountered. Electricity outages and problems with internet connectivity (47) might have affected participants in certain parts of the country, as Pakistan still has only 85 million 3G/4G subscribers and 40% internet coverage for a population of 0.2 billion people (48). Due to this, while not everyone was able to participate in the time-limited assessments, we wanted to make sure that no student got left behind in the learning component (our primary objective with the workshops). To tackle this head-on, our team posted summary handouts detailing major points discussed in the workshop. This was done only after the post-test submission was closed, to also ensure that the assessment results were reliable. Moreover, our sessions did not have one-to-one direct interaction between the participants and the presenters. Since brisk communication is a central to any teaching-learning activity (49), we anticipated that this may have interrupted rapport building and concomitantly affected the learning outcomes of a few participants. To counter this, we ensured that 20 organizers were available at every time to answer individual queries and troubleshooting concerns.

## Future Implications

As a long-term plan, our team has taken the initiative of establishing a nationwide undergraduate research network. Over 1,000 students from all over the country have expressed interest and sent applications to be research ambassadors at their home institutions. We hope that this workshop series was the steppingstone to establishing the first country wide undergraduate student research network.

## Conclusions

This workshop series establishes the first step in the development and feasibility testing of a curated virtual learning platform that has connected the undergraduate community in a low middle-income country. The overwhelming response seen with registrations (*N* = 3,862), particularly from students logging in from remote and rural areas of Pakistan, highlights the need to develop similar virtual workshops for medical students. It is imperative that this new-found momentum does not erode with time, and similar interventions are systematically launched to make undergraduate research understanding more accessible.

## Data Availability Statement

The raw data supporting the conclusions of this article will be made available by the authors, upon reasonable request.

## Ethics Statement

The study was reviewed and approved by Ethics Review Committee, Aga Khan University, Karachi. Digital informed consent was obtained from all students at the time of enrollment.

## Author Contributions

AN and MI have co-led the entire workshop series planning and execution at every stage since (and including) conception. They have both equally contributed in the writing of the manuscript and final revisions up until the last internal review. HS, HR, FA, FK, SA, AS, MM, and AM have actively participated in the conception, strategic planning, pilot, execution, data analysis and reporting, and manuscript preparation and final revisions. BH, MR, FJ, MT, and AH acted as expert supervisors and participated in the following: providing regular input during the planning and conception stages, assisting with the execution of the workshop series, reviewing and interpreting data, and preparing the manuscript up till the final revisions. AH and MT have led this series as principal investigator and co-investigator, respectively. All authors have read the final draft and approved it before submission.

## Funding

This study was supported by Medical College, Aga Khan University, Karachi, Pakistan.

## Conflict of Interest

The authors declare that the research was conducted in the absence of any commercial or financial relationships that could be construed as a potential conflict of interest.

## Publisher's Note

All claims expressed in this article are solely those of the authors and do not necessarily represent those of their affiliated organizations, or those of the publisher, the editors and the reviewers. Any product that may be evaluated in this article, or claim that may be made by its manufacturer, is not guaranteed or endorsed by the publisher.
