# Corrigendum: A Nationwide Virtual Research Education Program for Medical Students in Pakistan: Methodological Framework, Feasibility Testing, and Outcomes

**DOI:** 10.3389/fpubh.2022.926152

**Published:** 2022-05-11

**Authors:** Ali Aahil Noorali, Maha Inam, Hamna Shahbaz, Hareem Rauf, Faiqa Binte Aamir, Farah Khalid, Saadia Abbas, Abdullah Saeed, Muhammad Daniyal Musharraf, Asma Altaf Hussain Merchant, Babar S. Hasan, Muneera A. Rasheed, Fyezah Jehan, Muhammad Tariq, Adil Hussain Haider

**Affiliations:** ^1^Department of Medicine, Medical College, Aga Khan University, Karachi, Pakistan; ^2^Health Data Science Center, Clinical and Translational Research Incubator, Medical College, Aga Khan University, Karachi, Pakistan; ^3^Dean's Office, Medical College, Aga Khan University, Karachi, Pakistan; ^4^Medical College, Aga Khan University, Karachi, Pakistan; ^5^Department of Surgery, Washington University in St. Louis, St. Louis, MO, United States; ^6^Department of Paediatrics, Medical College, Aga Khan University, Karachi, Pakistan; ^7^Department for Educational Development, Medical College, Aga Khan University, Karachi, Pakistan; ^8^Department of Surgery and Community Health Sciences, Medical College, Aga Khan University, Karachi, Pakistan

**Keywords:** virtual course, research teaching, feasibility, medical students, public health

In the original article, there was a mistake in Figure 3 as published. While converting the figure file to TIFF, the incorrect file was converted and hence resulted in a mismatch between the text describing the figure and the figure itself. This error came to our notice post-publication, and we wanted to fix it as soon as possible, to ensure ethical conduct of research. The corrected Figure 3 appears below.

**Figure 3:**
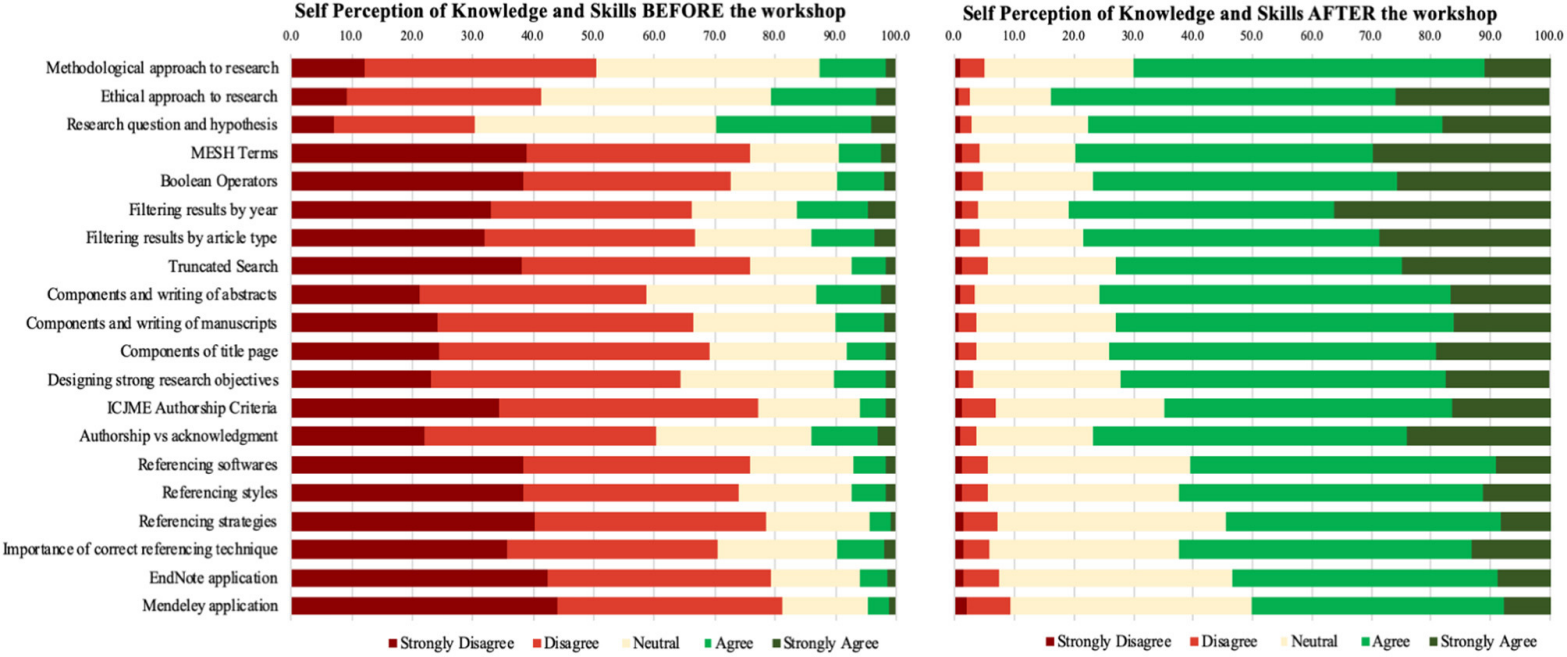
Subjective assessment: categorical assortment of participants' self-perceptions.

The authors apologize for this error and state that this does not change the scientific conclusions of the article in any way. The original article has been updated.

## Publisher's Note

All claims expressed in this article are solely those of the authors and do not necessarily represent those of their affiliated organizations, or those of the publisher, the editors and the reviewers. Any product that may be evaluated in this article, or claim that may be made by its manufacturer, is not guaranteed or endorsed by the publisher.

